# Risk of Posterior Capsular Rupture during Phacoemulsification in Patients with the History of Anti-VEGF Intravitreal Injections: Results from the Pan-American Collaborative Retina Study (PACORES) Group

**DOI:** 10.1155/2021/5591865

**Published:** 2021-10-11

**Authors:** Raul Velez-Montoya, Idaira Sanchez-Santos, Mauricio Galvan-Chavez, Lihteh Wu, J. Fernando Arevalo, María H. Berrocal, Arturo A. Alezzandrini, Marta S. Figueroa, Roberto Gallego-Pinazo, Rosa Dolz-Marco, Clara Martinez-Rubio, Roberto Gonzalez-Salinas

**Affiliations:** ^1^Retina Department, Asociación para Evitar le Ceguera en México IAP, México City 04030, Mexico; ^2^Asociados de Macula, Vitreo y Retina de Costa Rica, San Jose City 10102, Costa Rica; ^3^Retina Division, Wilmer Eye Institute, Johns Hopkins University School of Medicine, Baltimore, MD 21287, USA; ^4^Department of Ophthalmology, University of Puerto Rico, San Juan, PR 00907, USA; ^5^Ophthalmology Department, University of Buenos Aires, School of Medicine, Buenos Aires C1121, Argentina; ^6^Retina and Vitreous Department, Hospital Universitario Ramón y Cajal, Madrid 28034, Spain; ^7^Ophthalmology Department, Hospital Universitario y Politécnico La Fe, Valencia 46026, Spain; ^8^Anterior Segment Department, Asociación para Evitar le Ceguera en México IAP, DF 04030, México City, Mexico

## Abstract

**Purpose:**

To assess the risk for capsular rupture during routine phacoemulsification in patients with a history of anti-VEGF injections and other possible risk modifiers such as treatment patterns, type of anti-VEGF agent, and experience of the surgeon, among others.

**Methods:**

This study reviewed the medical records of 11,129 patients from 7 different hospitals in 5 countries. The study included 939 patients that underwent routine phacoemulsification and had a history of anti-VEGF therapy. We excluded patients with known risk factors for capsular rupture, as well as patients with a history of other retinal procedures. The study extracted data regarding general demographics, the number of previous injections, type of anti-VEGF agent, details of cataract surgery, and anti-VEGF treatment patterns.

**Results:**

Overall prevalence of posterior capsular rupture: 7.45% (95% CI: 5.9–9.32%). The mean number of injections per patient was 3.37 ± 2.8. More than 50% of the patients received their last anti-VEGF injection within three months before cataract surgery. The complication rate during intravitreal injections was 1.07%. In the univariate analysis, the experience of the cataract surgeon (inexperience surgeons; OR: 2.93) and the history of prior anti-VEGF therapy (OR: 1.77) were significant risk indicators for PCR (*p* < 0.05). However, after controlling for age in the multivariate analysis, the trend did not reach a statistical significance.

**Conclusion:**

The risk for capsular rupture is higher in patients with a history of intravitreal anti-VEGF injections.

## 1. Introduction

The identification of the vascular endothelial growth factor (VEGF) as the primary driver of choroidal neovascularization changed the treatment paradigm for all neovascular retinal diseases [1, 2]. This change led to the adoption and popularization of therapies based on intravitreal injections that may last for several years. As a result, intravitreal injections aimed at blocking VEGF have become the most performed ophthalmological procedure worldwide, exceeding by far any other surgical interventions [2, 3]. Although it is generally considered to be a low-risk procedure, it is not without adverse events [4]. The most common are those related to the injection procedure, such as conjunctival hemorrhages. The more serious adverse events include vitreous hemorrhage, retinal detachment, and endophthalmitis [4–6].

Increasing evidence suggests that a history of prior intravitreal injections increases the risk of posterior capsular rupture (PCR) during routine phacoemulsification [7–11]. Although the mechanism of capsular damage is still unknown, mechanical capsular damage via inadvertent posterior microtrauma by the injection, or even uncharacterized biochemical damage, has been mentioned as a potential cause. This evidence seems to suggest that the risk could be even higher for inexperienced surgeons and in patients with a history of more than ten injections [9, 11].

Because diseases that are usually treated with intravitreal injections have large periods of activity, it is not uncommon that a single patient may receive a significant number of injections in a single eye [12–14]. Furthermore, patients receiving intravitreal injections are usually elderly and may develop cataracts during follow-up. Therefore, it is important to further explore the association of intravitreal injections and PCR because some of these patients might be treated in teaching centers.

The aim of this study was to investigate the risk of PCR during routine phacoemulsification in patients with a history of intravitreal anti-VEGF injections. In addition, the risk was assessed according to other known concomitant relevant variables that could potentially ameliorate or magnify the risk, including an assessment of the type of intravitreal drug and treatment pattern.

## 2. Materials and Methods

This study was approved by each hospital's internal review board and was conducted according to the tenets of the Declaration of Helsinki and Good Clinical Practice guidelines. All sensitive data were managed according to Health Insurance Portability and Accountability Act (HIPAA) rules. Due to its retrospective design, an informed consent form was not required.

We manually reviewed all physical and electronic medical records of patients who underwent phacoemulsification and had a history of intravitreal injections from seven different hospitals and teaching centers in the United States, Puerto Rico, Mexico, Costa Rica, Argentina, and Spain. The surveyed period was ten years (January 2008 to December 2018).

From each medical record, the following data were extracted: general demographics (age and sex), number of previous intravitreal injections in the study eye, type of intravitreal drug used for each individual injection (bevacizumab (Genentech/Roche, San Francisco, CA, US), ranibizumab (Genentech/Roche/Novartis, San Francisco CA, US), and aflibercept (Regeneron/Bayer AG, Tarrytown, NY, US)), time (in months) between the last intravitreal injection and phacoemulsification (0–3 months, 3–6 months, and >6 months), existence of previously reported complications during any of the intravitreal injections (conjunctival hemorrhage, reported capsular damage, vitreous hemorrhage, and others), occurrence of PCR during phacoemulsification, and surgical experience of the performing surgeon. Surgical experience was classified according to each participating hospital training log, as inexperienced surgeons (less than 30 surgeries of experience), surgeons with an intermediate level of experience (31–250 surgeries of experience), and expert or experienced surgeons (more than 251 surgeries of experience).

The survey included only patients with a history of anti-VEGF injections. We excluded patients with a history of intravitreal steroids (triamcinolone, fluocinolone, or dexamethasone implant), intravitreal antibiotics, intravitreal ocriplasmin, pneumoretinopexy, and pars plana vitrectomy. Patients under 18 years of age, patients with known diagnosis of pseudoexfoliation, zonular dialysis, high myopia, a deep anterior chamber, or a known vitreous opacity (poor red reflex) at study initiation or with incomplete records were also excluded.

Statistical analyses were performed using Statistical Package for Social Sciences (SPSS) software (version 20, SPSS Inc., Chicago, IL, USA). Forest plots were generated using Prism GraphPad software (Prism Inc., version 8.0). Sample calculation was performed using a formula for proportion estimation considering a 2.22% incidence of PCR [9] in routine phacoemulsification surgeries in patients with a history of anti-VEGF injections, a confidence interval of 95%, a confidence limit of 1%, and a design effect of 1.0, *α* = 0.05, *β* = 0.20, and power 90%, for a minimum required sample of 826 subjects. Descriptive data are shown as the mean ± SD. Significance was assessed using Student's *t*-test and Mann–Whitney tests according to data distribution. Univariate analyses for significant risk indicators for PCR, including experience of the cataract surgeon, age, sex, type of anti-VEGF drug, and number of previous intravitreal injections, were performed by employing the chi-square test. In addition, multivariate logistic regression analysis was employed to assess the PCR risk indicators [15]. An alpha value < 0.05 was considered statistically significant. Bonferroni correction was used to adjust for the significance of the alpha value [16, 17]. The Gaussian distribution of all variables was determined using the D'Agostino–Pearson omnibus normality test.

## 3. Results

We reviewed a total of 11,124 medical records from which 939 (114% of the desired sample) belonged to patients who underwent routine cataract extraction, had a history of intravitreal anti-VEGF agents, and fulfilled all inclusion and exclusion criteria. Ninety-eight additional files were not included in the survey due to data inconsistency, incomplete description of the intravitreal injection procedure, and/or missing information regarding the anti-VEGF agents. This group comprised 504 females and 435 males, with a mean age of 64.58 ± 13.40 years at the time of surgery. From the total of entries in the sample, 70 procedures reported on file the occurrence of PCR. The overall prevalence of PCR was 7.45% (95% CI: 5.9–9.32%).

A total of 3,167 intravitreal injections were recorded from the files (3.37 ± 2.8 injections per patient). According to the treatment patterns, the group was divided further into four subgroups: (1) patients treated with bevacizumab exclusively, (2) patients treated with ranibizumab exclusively, (3) patients treated with aflibercept exclusively, and (4) patients treated with combination/switch therapy (patients in which treatment with a single anti-VEGF agent proved to yield an unsatisfactory response; thus, the treatment continued after changing the anti-VEGF agent).

Table 1 summarizes the general demographic data according to treatment pattern, including the number of injections per patient and type of drug, male/female distribution, mean age, and time between the last intravitreal injection and phacoemulsification surgery.

There were 10 (1.07%) reports of complications during the intravitreal injection procedure: five occurred in the bevacizumab exclusive group (one case of massive conjunctiva hemorrhage, one case of lens injury, and three cases of endophthalmitis), four occurred in the ranibizumab exclusive group (1 case of massive conjunctiva hemorrhage and 3 cases of vitreous hemorrhage that resolved spontaneously), and one occurred in the combination/switch therapy group (one case of lens injury). The overall prevalence of endophthalmitis was 0.09% (95% CI: 0.0905–0.099%). There were no other reports of lens capsule abnormalities, opacities, or suspected lesions after intravitreal injections during the preoperative examination before phacoemulsification.

**Figure 1:**
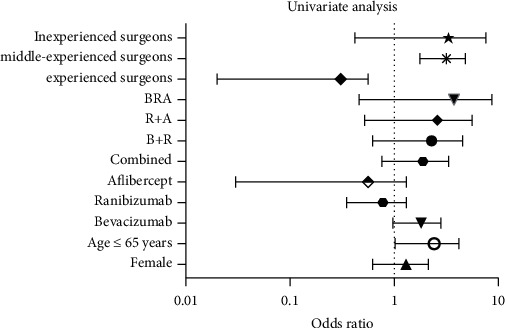
Predictors of posterior capsular rupture (PCR). The odds ratio with 95% confidence intervals is shown from the univariate analysis. The dotted line represents no difference in risk from the reference group within each predictor variable. The logarithmic notation was employed for scale representation of the odds ratios. Yr, years; BA, bevacizumab and ranibizumab; RA, ranibizumab and aflibercept; BA, bevacizumab and aflibercept; BRA, bevacizumab, ranibizumab, and aflibercept.

In the univariate logistic regression analyses, thirty-two of 435 males included in this study reported PCR. The overall odds ratio (OR) for male patients was 1.02 (95% CI: 0.62–1.66, *p*=0.9); whereas, 38 of 504 females reported PCR (OR: 0.97; 95% CI: 0.60–1.60, *p*=0.9) regardless of which anti-VEGF agent was employed. However, in the combination/switch therapy group, the overall OR for male patients (4 of 30) was 1.57 (95% CI: 0.45–5.38), but the difference did not reach statistical significance (*p*=0.7). An intermediate level of experience in cataract surgery (OR: 2.93) and a history of prior anti-VEGF therapy (OR: 1.77) were significant risk indicators for PCR (*p* < 0.05). There was no significant increase in the risk of PCR according to the type of intravitreal anti-VEGF agent or treatment pattern (Figure 1).

**Figure 2:**
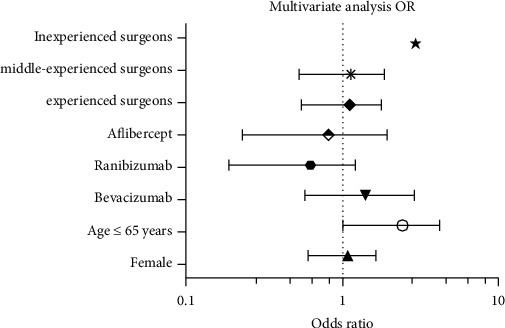
Predictors of posterior capsular rupture (PCR). The odds ratio with 95% confidence intervals is shown from multivariate logistic regression. The dotted line represents no difference in risk from the reference group within each predictor variable. The logarithmic notation was employed for scale representation of the odds ratios. Yr, years.

In the multivariate logistic regression model, older age (≤65 years) was a significant risk factor: OR: 2.0 (95% CI: 1.02–4.17, *p*=0.04). Considering the number of PCRs as a categorical variable, after adjusting for age (≤65 years), for patients receiving intravitreal injections of bevacizumab exclusively, the OR for PCR was 0.79 (95% CI: 0.58–2.83; *p*=0.56); for patients receiving intravitreal injections of ranibizumab exclusively, the OR for PCR was 0.47 (95% CI: 0.19–1.2; *p*=0.11); for patients receiving intravitreal injections of aflibercept exclusively, the OR for PCR was 0.23 (95% CI: 0.28–1.92; *p*=0.17); and for patients receiving combination/switch therapy with two or more drugs, the OR for PCR could not be determined due to an insufficient sample size. The ORs according to the level of experience of the cataract surgeon after adjusting for age were as follows: inexperienced surgeons: 2.94 (95% CI: 2.74–2.94; *p*=0.06), surgeons with an intermediate level of experience: 0.99 (95% CI: 0.53–1.85; *p*=0.98), and experienced surgeons: 0.98 (95% CI: 0.55–1.77; *p*=0.98) (Figure 2).

## 4. Discussion

Intravitreal injections of anti-VEGF agents have become the most commonly performed ocular procedure in the world. In 2016 alone, more than 3.5 million injections were performed in the US [9]. Although they are currently aimed at treating mainly retinal neovascular diseases, their use is expanding rapidly to other ocular diseases [18–20]. However, anti-VEGF therapy has two significant drawbacks: the first aims at treating chronic diseases with periods of activity that may last for several years and second, the short intravitreal half-life of the main available agents calls for repeated treatments to maintain efficacy [14, 21].

The safety profile of intravitreal injections has been comprehensively tested in several randomized clinical trials [4–6]. However, a local effect on anterior segment structures has seldom been studied. Data from several case reports, retrospective case series, and real-life experience studies have suggested increases in several risks associated with intravitreal injections, such as endophthalmitis, primary open-angle glaucoma, and surgical complications during cataract surgery (PCR) [7, 8].

For experienced surgeons, PCR during routine phacoemulsification is a rare event, with an incidence ranging from 0.45% to 3.6% [22, 23]. This surgical complication can occur during any step of the surgery and could lead to more severe complications (vitreous loss, lens fragment retention, retinal detachment, and endophthalmitis) [22]. In patients with a history of intravitreal injections, the risk of PCR is suspected to be significantly higher. A billing analysis from Medicare with a focus on claims of procedures associated with PCR occurrence during phacoemulsification (retained lens fragment removal (RLFR), anterior vitrectomy) showed that patients with a history of intravitreal injections had a 126% higher risk of needing such procedures (RLFR: hazard ratio 2.26; 95% CI: 1.19–4.30) [7]. In a later retrospective report, the same group reported a 3% prevalence of PCR among patients with a history of intravitreal injections, with an OR of 1.88 (*p* < 0.03) (sample: 197 patients). The authors also observed that patients with PCR tended to receive more previous injections than controls. However, the difference was not statistically significant [8]. Further evidence published in the same year (on larger samples) reported a 2.22% overall prevalence of PCR (95% CI 1.65–2.98; OR: 1.48–1.66) [9]. These authors successfully identified through multiple logistic regression models that the experience of the cataract surgeon (OR: 1.83–2.83), cataract progression grade (OR: 2.67–2.84), number of previous intravitreal injections (>10, OR: 2.59), and male sex (OR: 1.49) were potential associated risk factors for PCR [9, 10]. Finally, in early 2020, another group identified a dose-dependent increase of 8.6% (OR: 1.086, 95% CI: 1.04–1.135, *p* < 0.01) in relative risk per injection, especially in those receiving more than 10 injections [11]. Eventhough the evidence is compelling, there are some contradictions among the different studies. Nevertheless, all the findings suggest that the level of experience of the cataract surgeon seems to be a determinant-associated risk factor.

Our research was focused on patients with a history of anti-VEGF therapy. To assess the true prevalence of PCR, we excluded other types of intravitreal agents, such as steroids and antibiotics, mainly due to their known effect on the posterior lens capsule, as well as other ocular conditions, drugs, and procedures where there is a risk for unintentional posterior capsule damage that could go unnoticed during the preoperative workout. The prevalence of PCR observed in our patients with a history of intravitreal anti-VEGF therapy was 7.45% (95% CI: 5.9–9.32%). Despite this value representing the pooled prevalence from surgeons with various levels of experience, it is remarkable because it is significantly higher than that found in previous reports [9–11]. The reason for such an increase is unknown. The authors speculate that this might be due to selection bias introduced by the inclusion criteria, which yielded a sample with a high prevalence of retinal pathology. Moreover, patients who received more intravitreal injections were coincidentally the oldest (>70 years) in the sample, potentially skewing the results toward the higher end [24]. Although the prevalence of PCR in our sample remained high among experienced surgeons (6%), a high level of experience was in fact a protective factor, with a reduced OR of 0.34.

Our results further support the observations by Lee et al. [9] and Nagar et al. [11], who reported that the level of experience of the cataract surgeon and the age of the patient play a significant role in increasing the risk for PCR in patients with a history of intravitreal injections. In the univariate model, the risk was higher for inexperienced and surgeons with an intermediate level of experience. However, after controlling for patient age (>65 years) in the multivariate model, only the inexperienced surgeons maintained the trend toward a higher risk for PCR, but without reaching a statistical significance (*p*=0.06). The data in this research did not show any significant increase in the risk of PCR according to the type of intravitreal anti-VEGF agent used. Patients treated with bevacizumab exclusively showed a slightly increased OR, but the difference did not reach a statistical significance, and the trend disappeared in the multivariate logistic regression model. As in the data published by Shalchi et al. [10], we observed an increased risk for PCR regardless of the total number of previous intravitreal injections (OR: 1.77). Nevertheless, it is worth mentioning that our results did show that the group of patients on combination therapy had a higher prevalence of PCR (10–14%, data not shown) despite being performed by experienced surgeons. Unfortunately, the small number of patients who were receiving combination/switch therapy after cataract surgery prevented us from obtaining a sufficiently large sample to perform additional calculations. Therefore, this information should be interpreted with caution. The authors believe that patients receiving combination/switch therapy could reflect those with more severe, longer lasting, or unresponsive disease, which may lead to the need for more intravitreal injections (in this series, an average of 10.4 injections). It is unclear whether the repetitive insertion of a needle through the pars plana could exert some microtrauma over the lens capsule, which may lead to its debilitation and subsequent rupture during phacoemulsification, even without clear evidence of posterior capsule touch or lesion during any of the previous intravitreal injections.

Finally, in addition to its retrospective nature, this study has some limitations that we would like to acknowledge. The lack of a control group prevented us from comparing the prevalence of PCR in an age-matched group. However, this type of comparison was done by Lee et al., Shalchi et al., and Nagar et al. [9–11]. Instead, we focused exclusively on anti-VEGF therapy to assess the role of each agent in the risk of PCR. Nevertheless, due to the lack of treatment standardization across all the surveyed hospitals, the treatment patterns varied widely. There was a clear predominance of bevacizumab over the other two drugs. Therefore, the number of patients treated with ranibizumab and aflibercept was significantly smaller. This could have induced a type 2 error, and the OR could have been underestimated. Moreover, the predominance of bevacizumab therapy could suggest a sample obtained from a population with poor access to healthcare, which may have affected the quality of the cataract surgery available. Although the authors cannot explain the reason for this predominance nor draw any conclusion regarding socioeconomic variables from our population, it is important to highlight that all cataract surgeries were performed within the participant hospital facilities. Therefore, high-quality cataract surgeries with the best available technology were guaranteed for all the participants. The number of injections per patient in our sample was low. Only the combination therapy group reported an injection rate of 10 or more injections per patient. This could explain why we were not able to replicate the results of Lee et al. and Nagar et al. [11], regarding the number of previous intravitreal injections. Another limitation is the lack of sufficient information available regarding the intravitreal injection procedure. Although the medical records reported uneventful procedures, there is a possibility of underreported adverse events, changes in the intravitreal injection technique during the follow-up time, or that the injections were performed by a less experienced physician, all of which may increase the risk for inadvertent capsular damage. Finally, our study did not account for the cataract severity grade at the initiation of surgery or other known surgical technique-related risk factors for PCR, such as pupil and capsulorhexis diameters, which may have introduced a confounder in our analysis.

## 5. Conclusions

In summary, a history of previous anti-VEGF intravitreal injections is a significant factor for PCR in older patients. The multivariate analysis of the current data could not confirm any increase in the risk of PCR according to the type of the intravitreal anti-VEGF agent or treatment pattern. Although the results of the study suggest that there is a trend of a higher risk of PCR in inexperienced surgeons, the trend did not achieve a statistical significance (*p*=0.06). A larger sample is needed to explore this association further.

## Figures and Tables

**Table 1:** General demographic data.

	Bevacizumab	Ranibizumab	Aflibercept	Combination therapy	BR	RA	BA	BRA
Sample	598	231	39	89	35	26	14	14
Male	291	107	7	30	7	11	7	5
Female	307	106	32	59	28	15	7	9
Mean age ±SD	61.95 ± 12.84	67.80 ± 13.9	69.92 ± 13.56	72.2 ± 10.41	70.31 ± 11.48	76.19 ± 10.08	70.50 ± 11.18	71.21 ± 4.81
Last intravitreal injection (*N*/%)								
<3 months	306 (51.17%)	139 (65.26%)	22 (56.41%)	31 (34.83%)	5 (14.23%)	15 (57.69%)	5 (35.71%)	6 (42.86%)
3–6 months	88 (14.72%)	32 (15.02%)	8 (20.51%)	30 (33.71%)	19 (54.29%)	7 (26.92%)	2 (14.29%)	2 (14.29%)
>6 months	204 (34.11%)	42(19.72%)	9 (23.08%)	28 (31.46%)	11 (31.43%)	4 (15.38%)	7 (50.0%)	6 (42.86%)
Total number of injections	1378	726	134	929	254	332	124	216
Per patient overall ± SD	2.30 ± 3.34^*∗*^	3.41 ± 3.82^*∗∗*^	3.44 ± 3.37^*∗∗∗*^	10.44 ± 4.48				
				Per drug overall ± SD				
				Bevacizumab	5.11 ± 4.07		5.07 ± 2.97	7.64 ± 5.14
				Ranibizumab	2.14 ± 2.13	8.23 ± 6.13		3.64 ± 4.72
				Aflibercept		4.54 ± 4.38	3.79 ± 3.07	4.36 ± 3.48

BR, bevacizumab and ranibizumab; RA, ranibizumab and aflibercept; BA, bevacizumab and aflibercept; BRA, bevacizumab, ranibizumab, and aflibercept; SD, standard deviation; *N*/%, number of patients/percentages. ^*∗*^Interquartile range, 1–3; range, 1–52. ^*∗∗*^Interquartile range, 1–5; range, 1–24. ^*∗∗∗*^Interquartile range, 1–3; range, 1–13.

## Data Availability

The authors state that they have full control of all primary data, and the data are available from the corresponding author upon request. Access requests to sensitive data should be submitted in writing and should follow the tenets of the HIPAA rules and the current NOM at the moment of reception of the request. The local IRB will review any transfer of data. An institutional information transfer agreement may be needed. IRB Authorization: RE-19-03.

## Appendix

The Pan-American Collaborative Retina Study Group (PACORES) is an independent multinational study group, dedicated to the research, prevention, advancement of surgical techniques, and disease awareness of retinal diseases. The following investigators belong to the Pan-American Collaborative Retina Study (PACORES) Group: J. F. Arevalo (PI), T. Y. A. Liu, the Wilmer Eye Institute, Johns Hopkins University, Baltimore, Maryland, USA; L. Wu (PI), Asociados de Macula Vitreo y Retina de Costa Rica, San Jose, Costa Rica; A. F. Lasave (PI), Retina and Vitreous Service, Clinica Privada de Ojos, Mar del Plata, Buenos Aires, Argentina; Clinica Oftalmologica Centro Caracas and the Arevalo-Coutinho Foundation for Research in Ophthalmology, Caracas, Venezuela; M. Farah (PI), M. Maia, F. M. Penha, E. B. Rodrigues, Universidade Federal de São Paulo, Departamento de Oftalmologia, Instituto da Visão, São Paulo, Brazil; V. Morales-Canton (PI), J. Fromow-Guerra, J.L. Guerrero-Naranjo, J. Dalma-Weiszhausz, R Velez-Montoya, H. Quiroz-Mercado, Asociación para Evitar la Ceguera en Mexico, Mexico City, Mexico; F. J. Rodrigues (PI), F. E. Gomez, A. C. Brieke, A. Goveto, Fundacion Oftalmologica Nacional, Universidad del Rosario, Bogota, Colombia; M.H. Berrocal (PI), V. Cruz-Villegas, University of Puerto Rico, San Juan, Puerto Rico; F. Graue-Wiechers (PI), D. Lozano-Rechy, E. Fulda-Graue, Fundacion Conde Valenciana, Mexico City, Mexico; J.A. Roca (PI), A. Hernández, Clínica Ricardo Palma, Lima, Peru; M. J. Saravia (PI), A. Schlaen, J. Rojas, M. Ingolotti, Hospital Universitario Austral, Buenos Aires, Argentina; M. Avila (PI), L. Carla, Universidade Federal de Goiás, Departamento de Oftalmologia, Goiânia, Brazil; J. Cardillo (PI), R. Jorge, Hospital de Olhos de Araraquara and the Universidade de São Paulo, São Paulo, Brazil; C. Carpentier (PI), J. Verdaguer T., J.I. Verdaguer D., G. Sepúlveda, Fundacion Oftalmologica Los Andes, Santiago de Chile, Chile; A. Alezzandrini (PI), B. Garcia, M. Zas, OFTALMOS, Catedra de Oftalmologia, Universidad de Buenos Aires, Buenos Aires, Argentina; R. Gallego-Pinazo (PI), M. Diaz-Llopis, R. Dolz-Marco, J. R. García-Martínez, Clínica Oftalvist Valencia, Valencia, Spain; M. Figueroa (PI), I. Contreras, D. Ruiz-Casas, Hospital Universitario Ramón y Cajal, Departamento de Retina, and VISSUM Madrid, Madrid, Spain. ^*∗*^PI, principal investigator.
